# Copy Number Alteration Profiling from Plasma cfDNA WES in Advanced NSCLC

**DOI:** 10.3390/ijms262211111

**Published:** 2025-11-17

**Authors:** Ho Jang, Mi-Kyung Jeong

**Affiliations:** 1 Korea Medicine Data Division, Korea Institute of Oriental Medicine, Daejeon 34054, Republic of Korea; jh@kiom.re.kr; 2 Korea Medicine Convergence Research Division, Korea Institute of Oriental Medicine, Daejeon 34054, Republic of Korea

**Keywords:** plasma cell-free DNA, whole exome sequencing, copy number alteration detection, non-small cell lung cancer, GC bias

## Abstract

Circulating cell-free DNA (cfDNA) sequencing offers a minimally invasive approach for profiling tumor genomes, but detecting copy number alterations (CNAs) from cfDNA whole-exome sequencing (WES) remains technically challenging due to noise and guanine–cytosine (GC)-related bias. Building upon our previous study that characterized read count patterns in cfDNA WES data, we developed and evaluated an advanced pipeline for robust CNA detection in patients with advanced non-small cell lung cancer (NSCLC). Read count signals showed strong correlation with GC content, and applying locally estimated scatterplot smoothing (LOESS)-based GC bias correction effectively reduced false positives and improved CNA detection. The resulting cfDNA CNA profiles were reproducible within patients and showed strong concordance with The Cancer Genome Atlas (TCGA) tissue-level patterns for lung adenocarcinoma (LUAD) and lung squamous cell carcinoma (LUSC). These findings demonstrate that cfDNA WES, when combined with appropriate bias correction, can serve as a practical and minimally invasive alternative for genomic characterization of NSCLC.

## 1. Introduction

Recent advances in liquid biopsy technologies have enabled the analysis of tumor-derived materials such as circulating tumor DNA (ctDNA) and other cell-free DNA (cfDNA) fragments from blood and bodily fluids. These approaches have transformed cancer research and clinical practice by providing minimally invasive tools for early detection, monitoring of therapeutic response, and identification of emerging resistance mechanisms [1].

Among the diverse types of genetic alterations measurable in cfDNA, single nucleotide variants (SNVs) and copy number alterations (CNAs) represent two major classes of clinically relevant biomarkers. Their accurate detection enables comprehensive genomic profiling of tumors, offering insights into oncogenic driver events, tumor evolution, and therapy response. To quantify these alterations, two major methodological frameworks are commonly employed: droplet digital PCR (ddPCR) and next-generation sequencing (NGS).

ddPCR provides ultra-sensitive and quantitative detection of specific target mutations, making it ideal for applications where the genomic regions of interest are predefined. However, its targeted nature limits the breadth of genomic coverage, restricting discovery of novel or unexpected alterations. In contrast, NGS-based assays—including targeted panels, whole-exome sequencing (WES), and whole-genome sequencing (WGS)—allow for the simultaneous interrogation of thousands of genes, enabling both hypothesis-driven and exploratory biomarker analyses. NGS therefore adds value particularly in the context of comprehensive molecular characterization, where multiple genes and pathways must be evaluated from a limited cfDNA input.

Despite these advantages, NGS-based cfDNA analysis poses unique technical challenges. Because cfDNA is typically fragmented and available in low quantities, PCR amplification is required during library preparation. This process can introduce errors that compromise the accuracy of variant detection, leading to false-positive SNVs or biased estimates of copy number and allele frequency [1]. Several strategies have been developed to mitigate these issues, such as molecular barcoding to identify PCR duplicates, and the inclusion of matched normal controls to distinguish somatic from germline variants and to correct amplification artifacts [2]. However, such approaches may increase experimental cost and complexity, limiting their feasibility in routine clinical practice.

Whole-exome sequencing (WES) has emerged as a practical compromise between sensitivity, breadth, and cost. It focuses on protein-coding regions that harbor the majority of actionable mutations and has demonstrated reasonable performance in detecting CNAs and SNVs. When paired tumor-normal analyses are available, WES can provide accurate somatic mutation profiles; yet, obtaining matched normal DNA remains a logistical and financial barrier in many clinical settings [3].

Previously, we explored the feasibility of CNA detection from cfDNA WES data of 40 stage IV non-small cell lung cancer (NSCLC) patients, even in the absence of matched normal controls [4]. In that study, we proposed a read count-based normalization approach that effectively reduced technical noise and enabled detection of recurrent CNAs in major lung cancer-related genes. While this demonstrated the potential of cfDNA WES for CNA profiling, systematic biases—particularly GC-content-related artifacts—remained a limiting factor for reliable detection.

In the present study, we sought to refine and extend our previous framework to improve the robustness and biological interpretability of cfDNA-derived CNA profiles. We expanded the patient cohort, implemented GC bias correction within the read count normalization pipeline, and evaluated reproducibility by analyzing serial plasma samples from individual patients. Finally, we compared cfDNA-derived CNA landscapes with The Cancer Genome Atlas (TCGA) lung adenocarcinoma (LUAD) and lung squamous cell carcinoma (LUSC) datasets to assess concordance between cfDNA and tissue-based genomic profiles. Through this work, we aim to establish an analytically enhanced and biologically validated strategy for cfDNA WES-based CNA detection, demonstrating its potential as a practical and minimally invasive alternative to tissue genomic profiling in advanced NSCLC.

## 2. Results

### 2.1. Sample Characteristics

A total of 170 patients were enrolled during the study period. Among the enrolled patients, 18 individuals had at least two plasma samples that passed cfDNA quantity/quality control (QC). Successful library preparation and sequencing were completed for 15 patients. During follow-up, 14 patients (93%) experienced progression as assessed by the Response Evaluation Criteria in Solid Tumors (RECIST) version 1.1, with a median progression-free survival (PFS) of 63 days (95% CI, 56–126). These clinical outcomes provide context for evaluating CNA detection performance, although the study was not designed to assess clinical efficacy. For these 15 patients, a total of 31 plasma cfDNA WES samples with sufficient read coverage in the target regions were included in the final analysis. The minimum input amount of cfDNA among the sequenced samples was 10 ng, with a median of 29 ng.

For healthy subjects, eight out of ten individuals with sufficient read coverage were included in the analysis.

### 2.2. Relationship Between Read Count Patterns and GC Content

In the previous study, we observed that the read count pattern of bins in cfDNA WES data was similar among NSCLC samples [4]. These results are consistent with the cfDNA WES datasets from normal subjects in this study. The average Pearson correlation coefficient for all pairs of eight normal samples was r=0.947, and all $p<2.2\times 10^{-16}$, implying that independent normal subjects also share a high level of similarity.

**Figure 1:**
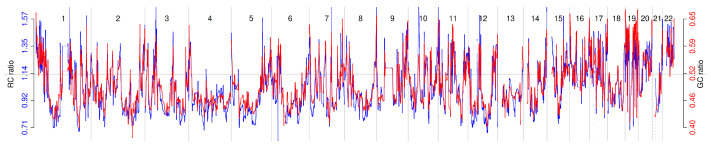
Genome-wide Read Count and GC Ratio Signals. The following highlights are the same. The blue line represents the normalized read count (NRC), and the red line represents the GC ratio.

Figure 1 visually shows that the averaged read count pattern (blue) derived from eight normal subjects, each with a median read count greater than 30, resembles the GC ratio pattern (red) of the same bins. The left y-axis of the figure represents the normalized read count, while the right y-axis indicates the GC ratio. These signals were denoised using a translation-invariant (TI) wavelet transform. The Pearson correlation coefficient between the two denoised signals was r=0.776 ($p<2.2\times 10^{-16}$), implying that a portion of the read count pattern reflects differences in GC ratios across genomic regions.

### 2.3. Residual GC Bias Correction in Normal Subjects

**Figure 2:**
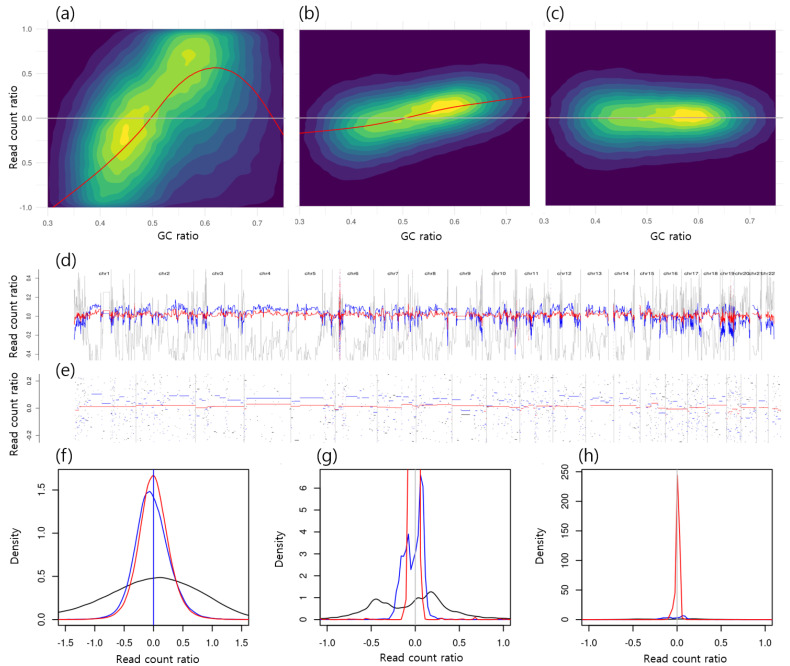
Visualization of bias embedded in the normalized read count (NRC) and the results of its correction. (**a**) NRC of a normal sample without a matched control. Brighter colors indicate bins with higher density, whereas darker colors indicate bins with lower density. The red curve represents the LOESS-fitted NRC according to the GC ratio. (**b**) NRC of the sample normalized by a control. (**c**) NRC of the sample normalized by a control with GC bias correction applied. (**d**) Denoised NRC for the three cases: gray line represents (**a**), blue line represents (**b**), and red line represents (**c**). (**e**) Segmented NRC for the three cases: the gray line corresponds to (**a**), the blue line corresponds to (**b**), and the red line corresponds to (**c**). (**f**) Distribution of NRC for the three cases: black indicates (**a**), blue indicates (**b**), and red indicates (**c**). (**g**) Distribution of segmented NRC for the three cases: black, blue, and red correspond to the gray, blue, and red segments shown in (**e**), respectively. (**h**) Expanded view of (**g**), showing a more centralized red distribution around zero compared with the others.

Figure 2a shows the two-dimensional distribution of bins in a normal subject, plotted as normalized read count (NRC) versus GC ratio. This corresponds to the denoised NRC signal in gray in Figure 2d and the short gray segments in Figure 2e, indicating systemic bias and potential false positives in CNA detection. The black curve in Figure 2f displays a dispersed pattern of raw NRC signals (median NRC=0.000, IQR −0.593to0.535), while the multimodal black distribution in Figure 2g represents segmented NRC bins (median NRC=−0.006, IQR −0.402to0.219).

In our previous study [4], read count fluctuations were reduced by dividing the raw read count of a test sample by that of a control sample. Figure 2b shows the distribution of NRC when normalizing one normal subject by another, where a correlation between GC ratio and NRC still remained. This corresponds to the denoised NRC signal in blue in Figure 2d and the blue segments in Figure 2e, suggesting persistent systemic bias and false positives in CNA detection. The blue curve in Figure 2f indicates a more centralized distribution of normalized NRC signals (median NRC=−0.037, IQR −0.211to0.156), though the center of the distribution does not align exactly at zero. Similarly, the blue curve in Figure 2g represents segmented NRC bins that are more centralized (median NRC=−0.005, IQR −0.104to0.071) than those of raw NRC but still incompletely corrected.

After applying GC bias correction, the absolute values of the correlation coefficients decreased across all sample pairs. Figure 2c illustrates the relationship between GC ratio and NRC after GC bias correction using the same pair of normal subjects. This corresponds to the denoised NRC signal in red in Figure 2d and the red segments in Figure 2e, demonstrating reduced systemic bias and fewer false positives in CNA detection. The red curve in Figure 2f shows a more centralized distribution of GC-corrected NRC signals (median NRC=0, IQR −0.162to0.166), with its mean closer to zero. Likewise, the red distribution in the segmented NRC bins in Figure 2g is more centralized (median NRC=0.003, IQR −0.011to0.019) compared with those of the raw NRC. Figure 2h presents a broader view of Figure 2g, illustrating that the red density distribution is more centralized around zero compared with the others.

**Figure 3:**
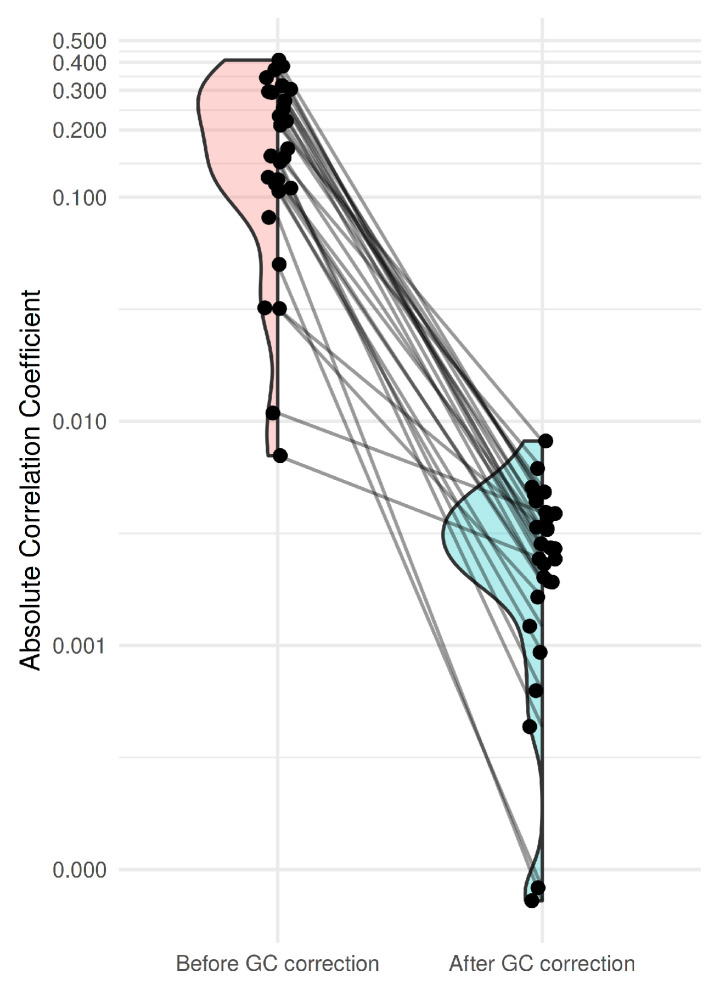
Changes in correlation coefficients between normalized read count (NRC) and GC ratio before and after GC bias correction. Red distribution represents the absolute correlation coefficients between NRC and GC ratio before applying GC bias correction, while blue distribution represents those after correction. Lines connecting the points indicate the same pair of normal subjects.

Figure 3 presents the distribution of correlations between the NRC and GC ratio before and after GC bias correction using LOESS fitting. For each pair of normal subjects, correlations between NRC and GC ratio were calculated to assess residual GC-related bias. Prior to correction, the absolute correlation was median |ρ|=0.159 (IQR 0.109–0.294) across all normal-pair comparisons; after correction, it decreased to 0.003 (IQR 0.002–0.004) (paired Wilcoxon, $p=7.451\times 10^{-9}$). These results demonstrate that GC bias correction substantially reduced GC-related artifacts, as the correlations between NRC and GC ratio after correction were consistently lower than those observed before correction across all sample pairs. Both the test and control datasets were derived from normal subjects, under the assumption that no CNAs were present.

Taken together, these results indicate that read count signals are influenced by GC bias, and that applying GC bias correction effectively reduces residual systematic bias after normalization, thereby improving the accuracy of CNA detection.

### 2.4. CNA Detection and Comparison of Aggregated CNA Profiles Between Cancer Types

To assess the biological relevance of our CNA detection pipeline, we compared cfDNA WES-derived CNA profiles from NSCLC patients with tissue-based reference datasets from TCGA. This analysis was designed to evaluate whether plasma-derived genomic signals capture cancer type-specific CNA patterns consistent with those observed in tumor tissues, thereby validating the biological relevance of cfDNA-based CNA detection.

**Figure 4:**
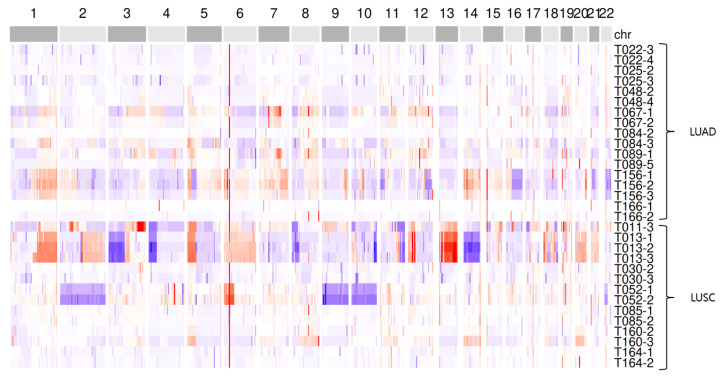
Heatmap of GC bias-corrected CNA segmentations in LUAD and LUSC subjects. Red indicates copy number gains, and blue indicates copy number losses. Chromosome numbers are shown along the top of the heatmap, and sample identifiers are displayed along the right side.

We applied our correction methods to 31 plasma WES samples obtained from 15 advanced NSCLC patients, including 8 with LUAD and 7 with LUSC. Figure 4 presents genome-wide CNA patterns after GC bias correction in the form of a heatmap. In the sample code T*i*-*j*, *i* denotes the subject identifier and *j* indicates the visit number, where j=1 corresponds to the first visit (baseline). CNA profiles from multiple samples of the same patient displayed high intra-patient similarity (median Spearman’s ρ=0.67, IQR 0.60to0.77), supporting the reproducibility of the detection pipeline.

The aggregated CNA signals from our LUAD and LUSC cfDNA samples were then compared with those from 32 TCGA cancer types. The CNA segment datasets for all 32 TCGA cancer types were obtained from the Firehose Legacy collection via cBioPortal [5], and Spearman’s correlation coefficients were calculated using 0.5Mbp bins between our dataset and the aggregated CNA profiles of each TCGA cancer type.

**Figure 5:**
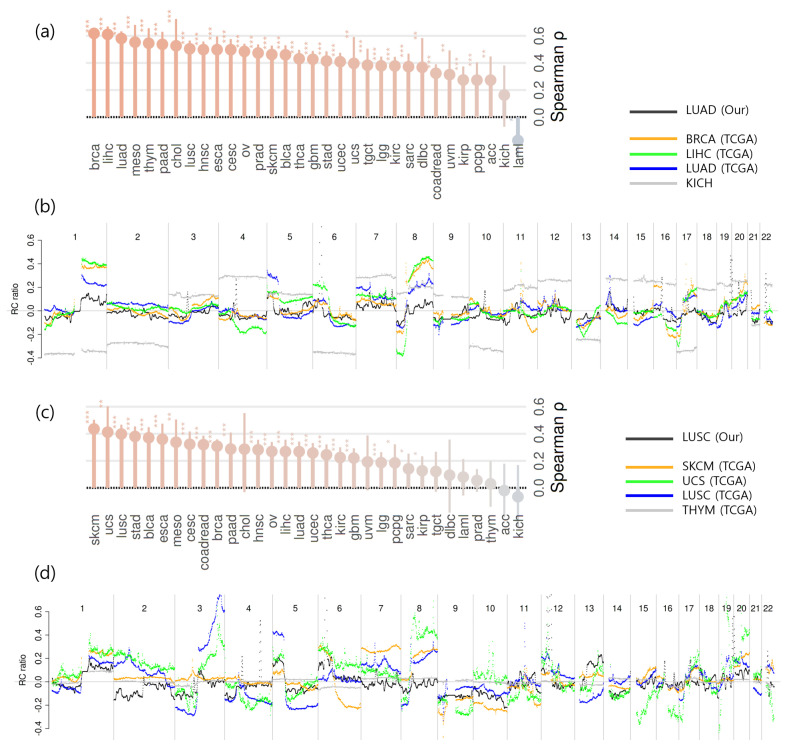
Correlation of aggregated cfDNA WES CNA profiles with TCGA cancer types. (**a**) Rankings of correlation coefficients between the aggregated CNA profile of our LUAD samples and those of 32 TCGA cancer types. (**b**) Aggregated CNA profiles of our LUAD cohort compared with the top three TCGA cancer types (BRCA, LIHC, and LUAD) and the lowest-ranked type (KICH). (**c**) Rankings of correlation coefficients between the aggregated CNA profile of our LUSC samples and those of 32 TCGA cancer types. (**d**) Aggregated CNA profiles of our LUSC cohort compared with the top three TCGA cancer types (SKCM, UCS, and LUSC) and the lowest-ranked type (THYM). Statistical significance between the aggregated CNA profiles of our cohort and those of 32 TCGA cancer types was determined using Fisher’s z-transformed Spearman correlations with Benjamini–Hochberg FDR correction. Significance levels: * q≤0.05; ** q≤0.01; *** q≤0.001.

Figure 5a shows the ranking of correlation coefficients between the aggregated CNA profiles of our LUAD cohort and those of TCGA cancer types. For LUAD, the top three correlations were BRCA (ρ=0.617), LIHC (ρ=0.610), and TCGA LUAD (ρ=0.580), with a gap to the fourth-ranked cancer type of Δρ=0.027. Although LUAD was not the single highest, it was ranked among the top three, indicating a strong correspondence between our cfDNA-based LUAD profile and tissue-level CNAs observed in TCGA.

Similarly, Figure 5c presents the correlation ranks between our LUSC aggregated profile and TCGA cancers, where the top three were SKCM (ρ=0.435), UCS (ρ=0.411), and TCGA LUSC (ρ=0.398), with Δρ=0.018 to the fourth. Figure 5b,d further visualize the genome-wide CNA patterns of our aggregated cfDNA signals alongside TCGA reference profiles for LUAD and LUSC, respectively. These findings indicate that cfDNA WES-derived CNA profiles can recapitulate tissue-level CNA landscapes of LUAD and LUSC. While the strongest correlations were not always with the same histologic subtype, our LUAD and LUSC profiles consistently ranked among the top three across the 32 TCGA cancer types, suggesting overall concordance with tissue-level genomic patterns despite minor inter-cancer variability.

### 2.5. Identification of LUAD- and LUSC-Related Genes Within CNA Regions

To determine whether the cfDNA WES-based CNA profiles capture biologically and clinically meaningful alterations, we examined whether known LUAD- and LUSC-related driver genes were represented within the detected CNA regions. Establishing the concordance between cfDNA-derived CNAs and well-characterized cancer genes is essential to validate the biological relevance and potential clinical utility of cfDNA WES for noninvasive tumor genotyping.

**Figure 6:**
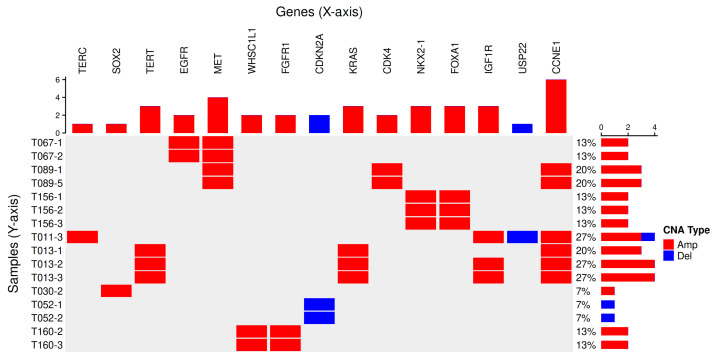
Copy number alterations involving LUAD- and LUSC-related genes. Known NSCLC genes affected by copy number alterations and their detection frequencies across samples are shown along the top of the figure. The proportion of altered genes in each sample is shown along the right side. Red indicates copy number gains, and blue indicates copy number losses.

We investigated CNA segments exceeding the detection threshold calculated using the method described in our previous study [4]. A total of 33 LUAD genes and 42 LUSC genes reported as focally gained or deleted in the pan-cancer study by the TCGA Research Network [6] were used as reference lists to assess gene-level alterations in our dataset. Figure 6 illustrates LUAD- and LUSC-related genes observed within CNA regions in our cfDNA WES samples.

**Figure 7:**
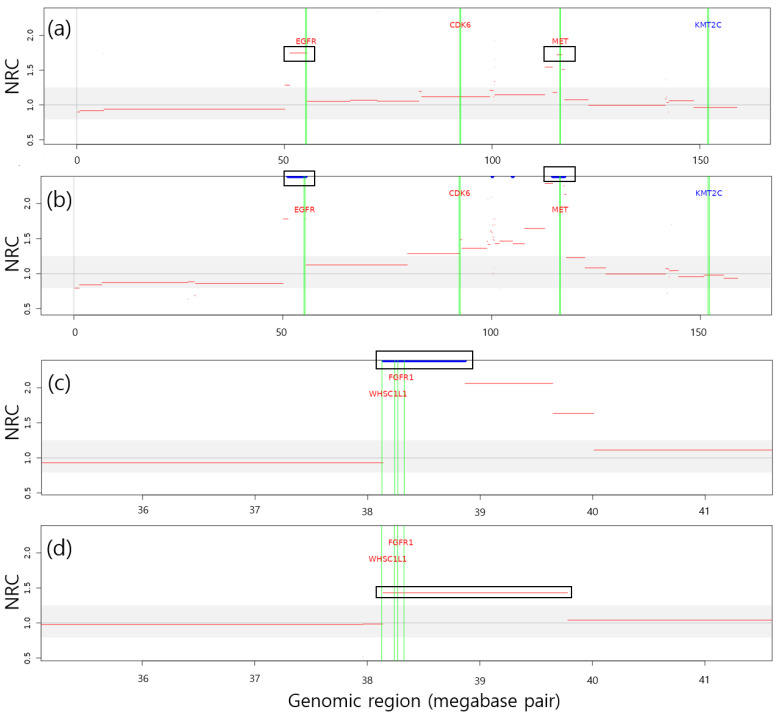
Examples of NSCLC-related genes detected within CNA segments. (**a**,**b**) Copy number gains involving *EGFR* and *MET* genes on chromosome 7 in samples T067-1 and T067-2, respectively. (**c**,**d**) Copy number gains involving *WHSC1L1* and *FGFR1* genes on chromosome 8 in samples T160-2 and T160-3, respectively. Each red segment represents the copy number state along genomic regions. Black rectangles mark confirmed CNA segments encompassing NSCLC-related genes. Green vertical lines indicate the genomic positions of the candidate genes. Blue segments denote CNAs whose values exceed the upper limit of the figure. Segments within the light gray area fall below the detection threshold and are considered neutral regions.

In total, 39 LUAD- and LUSC-related genes were detected among 16 cfDNA samples from 8 subjects. Amplifications involving *EGFR*, *MET*, *CDK4*, *NKX2-1*, *FOXA1*, and *CCNE1* were identified in 3 of 8 LUAD patients. These genes were consistently detected across all samples obtained from those individuals. In LUSC patients, *REL*, *TERC*, *SOX2*, *TERT*, *WHSC1L1*, *FGFR1*, *CDKN2A*, *KRAS*, *IGF1R*, *USP22*, and *CCNE1* were observed in 5 of 7 subjects. Except for two subjects (T011 and T030), all remaining subjects showed consistent CNA involvement of these genes in at least two longitudinal samples. A single discrepancy was noted for the *IGF1R* gene in sample T013-1, which did not exceed the detection threshold; however, visual inspection revealed CNA patterns comparable to those in T013-2 and T013-3. Figure 7 illustrates representative CNA regions encompassing NSCLC-related genes consistently identified in two patients.

### 2.6. Effect of GC Bias Correction on CNA Detection Performance

Accurate correction of GC bias is critical for enhancing the reliability and efficiency of CNA detection in cfDNA WES data, as residual GC-related artifacts can lead to false positives and obscure true genomic alterations. To quantify this improvement, we assessed CNA detection performance before and after GC bias correction by measuring the total inspection length (in base pairs) and the total number of CNA segments required to detect the 39 LUAD- and LUSC-related genes described in the previous section. CNA segments were examined in descending order of absolute signal value, from the most pronounced to those closest to the neutral CNA state.

For example, to identify the *CCNE1* amplification in sample T089-1, a total genomic span of 260.2Mbp across 1708 segments needed to be inspected without normalization using a control sample, 38.0Mbp across 386 segments with normalization but before GC bias correction, and only 27.6Mbp across 99 segments after correction.

**Figure 8:**
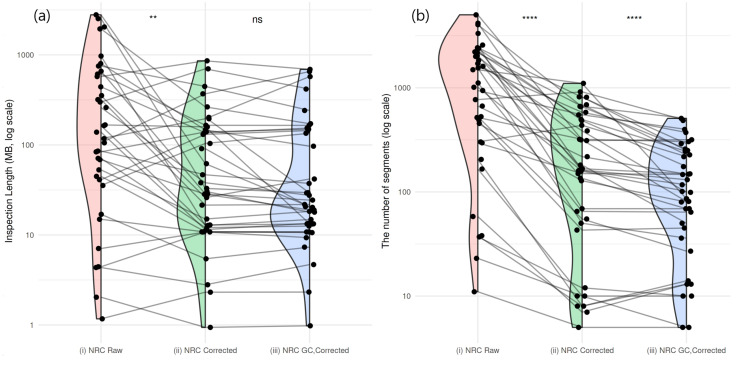
Comparison of gene detection cost before and after GC bias correction. (**a**) Detection cost measured by the total inspection length. ‘NRC Raw’ (red) indicates results without a control sample. ‘NRC Corrected’ (green) indicates results using a control sample. ‘NRC GC-Corrected’ (blue) indicates results using a control sample with additional GC bias correction. (**b**) Detection cost measured by the number of segments. The statistical significance between two groups is indicated as follows: ns, not significant (p>0.05); ** p≤0.01; **** p≤0.0001.

Figure 8a shows changes in detection performance, measured by inspection length, for 38 of the 40 genes detected across all three analysis settings: (i) normalization without a control sample (red), (ii) using a control sample without GC bias correction (green), and (iii) using a control sample with GC bias correction (blue). The median inspection lengths were 138.58Mbp (IQR 42.92to593.59) for $NRC_{\mathrm{raw}}$, 32.89Mbp (IQR 12.06to146.65) for $NRC_{\mathrm{corrected}}$, and 21.212Mbp (IQR 13.24to142.03) for $NRC_{\mathrm{GC},\mathrm{corrected}}$. Overall, inspection lengths decreased significantly from $NRC_{\mathrm{raw}}$ to $NRC_{\mathrm{corrected}}$ ($p=3.638\times 10^{-3}$), whereas the decrease from $NRC_{\mathrm{corrected}}$ to $NRC_{\mathrm{GC},\mathrm{corrected}}$ was not statistically significant (p=0.1569).

Figure 8b presents the corresponding changes in the number of inspected segments for the same set of genes. The median segment counts were 1113 (IQR 378to2187) for $NRC_{\mathrm{raw}}$, 167 (IQR 55to496) for $NRC_{\mathrm{corrected}}$, and 100 (IQR 46to240) for $NRC_{\mathrm{GC},\mathrm{corrected}}$. The reductions from $NRC_{\mathrm{raw}}$ to $NRC_{\mathrm{corrected}}$ ($p=1.201\times 10^{-6}$) and from $NRC_{\mathrm{corrected}}$ to $NRC_{\mathrm{GC},\mathrm{corrected}}$ ($p=2.934\times 10^{-6}$) were both statistically significant (Wilcoxon signed-rank test, paired). Together, these results demonstrate that GC bias correction effectively reduces false-positive segments and improves CNA detection efficiency.

## 3. Discussion

We systematically evaluated the sources of technical bias and biological relevance in cfDNA WES-based CNA detection. Our findings revealed that GC content strongly influences read count patterns, and that GC bias correction effectively reduces systematic artifacts, improving CNA detection accuracy. Furthermore, cfDNA-derived CNA landscapes closely mirrored tissue-level profiles and captured known LUAD- and LUSC-related driver genes, supporting the biological and clinical relevance of cfDNA WES as a minimally invasive approach for CNA profiling in NSCLC.

While these results demonstrate the feasibility of reliable CNA detection from cfDNA WES data that pass quality control, several technical and analytical challenges remain. Addressing these issues will further enhance the robustness and clinical utility of cfDNA WES-based CNA analysis.

### 3.1. Improvement of CNA Detection Performance

Using our multi-step analytical pipeline, we successfully reduced GC bias-related false positives in CNA detection. However, some residual artifacts were not fully corrected by the GC bias adjustment implemented in this study. The one-dimensional relationship between GC content and read count was corrected using LOESS modeling, but future work could apply more flexible approaches capable of capturing multidimensional dependencies. For example, incorporating additional genomic variables such as mappability [7] may further improve accuracy by reflecting local genomic characteristics.

### 3.2. Enhancing Detection Sensitivity and Threshold Adaptation

Applying a conservative detection threshold allowed us to identify CNAs likely associated with cancer. Visual inspection revealed CNA-like patterns even in some samples that did not meet the threshold criteria. Although these CNA segments were excluded due to low ctDNA content and limited reliability, they still appeared to provide biologically meaningful information. Future studies could explore adaptive or ctDNA fraction-adjusted thresholds to optimize sensitivity while minimizing false positives. Additionally, ranking CNAs by their relative size or signal strength could provide another dimension for integrating subthreshold information.

### 3.3. Challenges in Detecting CNAs from cfDNA WES

While lung adenocarcinoma (LUAD) and lung squamous cell carcinoma (LUSC) tissues typically harbor numerous CNAs, several stage IV cases in our cohort showed no detectable CNAs in cfDNA WES data. This observation can be explained by two primary factors. First, CNA detection requires a sufficiently high circulating tumor DNA (ctDNA) fraction; when ctDNA levels fall below approximately 10%, the copy number signal is often obscured by the predominance of normal cfDNA [8]. Second, technical limitations of cfDNA WES—such as the short and variable fragment size of cfDNA—can reduce the reliability of read-depth-based CNA detection [8]. Previous large-scale tissue studies, including TCGA, have demonstrated that LUAD and LUSC carry a substantial CNA burden; for example, the TCGA LUSC cohort reported an average of more than 300 copy number segments per tumor [9]. Therefore, the apparent absence of CNAs in certain cfDNA WES samples most likely reflects low ctDNA content and inherent technical constraints of cfDNA sequencing, rather than a genuine lack of CNAs in the corresponding tumors.

### 3.4. Clinical Relevance of CNA-Affected Genes in LUAD and LUSC

We identified recurrent copy number alterations involving well-known oncogenic drivers and tumor suppressors to evaluate the utility of plasma cfDNA WES data in LUAD and LUSC. For this analysis, we focused on cancer genes listed in the TCGA pan-cancer study [6], which provides key information for NSCLC patients. The clinical significance of the copy number-altered cancer genes identified in our cohort is summarized as follows.

Copy number gains near the *TERC* gene were identified in two patients, each with a single sample. *TERC* (3q26.3) frequently exhibits copy number gain in NSCLC/LUSC, including high-grade preinvasive bronchial squamous lesions. Increased *TERC* dosage is associated with elevated expression and enhanced telomerase activity in lung cancer [10,11].

Copy number gains of *SOX2* were observed in one LUSC patient. This alteration is a common driver in LUSC and supports tumor growth [12], but in our dataset it showed no direct link to clinical outcomes with immune checkpoint inhibitor therapy.

Copy number gains around the *TERT* gene were found in one patient, showing consistent gains across multiple samples. Arm-level 5p gains including *TERT* (5p15.33) are characteristic of NSCLC and appear even in early-stage disease [13]. Functionally, *TERT* copy number gain correlates with higher mRNA expression and poorer prognosis in NSCLC [14].

Focal amplifications of the *EGFR* gene were consistently detected in one patient across two samples. The presence of this alteration, previously confirmed as an *EGFR* mutation before treatment, suggests that WES can provide complementary information on the genomic landscape of the tumor. In stage IV NSCLC patients treated with atezolizumab, *EGFR* focal amplification—often associated with resistance to EGFR-TKI therapy—may indicate a tumor phenotype less responsive to PD-L1 blockade [15].

Focal amplification of the *MET* gene was consistently identified in two patients. Although *MET* amplification has not been linked to improved benefit from PD-1/PD-L1 blockade in a prospective cohort [16], other studies suggest that high *MET* copy number can attenuate the efficacy of immune checkpoint inhibitors by suppressing antitumor immune signaling [17].

In one subject, CNA gains around *WHSC1L1* and *FGFR1* were consistently detected in two samples. *FGFR1* amplification is a recurrent alteration in NSCLC, particularly in squamous carcinoma, and confers oncogenic dependency targetable by FGFR1 inhibitors [18]. NSD3 (*WHSC1L1*), a histone H3K36 methyltransferase, acts as a key oncogenic driver of the 8p11–12 amplicon in LUSC and promotes tumor progression [19]. In NSCLC, *FGFR1* amplification shows inconsistent clinical relevance for immune checkpoint therapy, whereas *WHSC1L1* amplification has been associated with an immune-desert phenotype [20].

Copy number loss of *CDKN2A* was consistently observed in one patient across two samples. In NSCLC, *CDKN2A* loss-of-function correlates with worse outcomes under PD-1/PD-L1 inhibitor therapy, including atezolizumab [21].

*KRAS* focal amplification was consistently detected in one patient. Although uncommon in NSCLC, *KRAS* focal amplification can act as an independent oncogenic driver and is linked to tumor progression and poor prognosis, while its influence on PD-1/PD-L1 inhibitor response remains unclear [22].

*CDK4* focal amplification was also consistently observed in one patient. In NSCLC, *CDK4* amplification disrupts the RB pathway and correlates with poor prognosis, reflecting aggressive tumor biology [23]. Although its predictive value for atezolizumab efficacy is uncertain, *CDK4* activation has been linked to an immunosuppressive tumor microenvironment, suggesting potential resistance to PD-L1 blockade [24].

CNA gains around *NKX2-1* and *FOXA1* were consistently observed in one subject across three samples. In lung adenocarcinoma, *NKX2-1* (TTF-1) gain at 14q13.3 acts as a lineage-survival oncogene essential for tumor growth [25], but evidence does not support a direct relationship with immune checkpoint inhibitor response. *FOXA1* copy number gain has been linked to poorer overall survival in clinical cohorts [26], but no direct evidence connects it to immunotherapy outcomes.

CNA gains around *IGF1R* were observed in two LUSC subjects. In advanced NSCLC, *IGF1R* expression is common, particularly in squamous carcinoma, but it has not shown a significant association with overall survival, suggesting that *IGF1R* gain or expression alone is not a prognostic marker [27].

CNA loss around *USP22* was observed in one LUSC subject. *USP22* has been reported as a recurrent focal deletion in LUSC, though its functional role in this context remains unclear.

Focal amplification of *CCNE1* was observed in three patients, with two showing consistent results across samples. In NSCLC, *CCNE1* (Cyclin E1) focal amplification indicates hyperactivation of the Cyclin E–CDK2 axis and aligns with evidence that Cyclin E overexpression is a negative prognostic factor in lung cancer [28]. For immunotherapy (atezolizumab/pembrolizumab), *CCNE1* is not an established predictive biomarker; current reviews suggest that only PD-L1 expression, deficient MMR, and high TMB are validated predictors for PD-1/PD-L1 blockade [29].

Taken together, these results indicate that cfDNA WES can reproduce tissue-level CNA landscapes, supporting its feasibility as a complementary and minimally invasive approach for genomic characterization of NSCLC.

### 3.5. Limitations in Linking CNA Signals to Clinical Outcomes

We restricted the analysis to subjects with at least two plasma cfDNA samples that passed quality control (QC), including those categorized as ‘hold’. Except for a single case, most participants experienced progressive disease (PD) before study completion. Although the only non-PD subject showed no detectable CNAs, the pronounced imbalance between PD and non-PD groups, along with the small cohort size, limited the statistical power to compare genomic differences between clinical outcomes.

Previous studies have reported that the presence of CNAs is associated with poorer responses to immunotherapy, and in our cohort, all subjects with CNA-positive samples showed disease progression. However, a subset of PD subjects did not exhibit detectable CNAs. One plausible explanation is a low tumor fraction (TF) in those samples, which may have hindered CNA detection. Alternatively, CNAs alone may not fully capture the molecular mechanisms underlying progression.

To enhance explanatory power, future analyses incorporating single-nucleotide variants (SNVs) and other genomic alterations are warranted. However, in the absence of matched normal controls, it remains challenging to draw definitive conclusions from SNVs identified in plasma cfDNA. Accordingly, future studies should aim to include matched normal samples or implement analytical strategies to minimize false positives in SNV detection when matched normals are unavailable.

## 4. Materials and Methods

### 4.1. Patients, Samples, and WES Procedures

Patients with advanced non-small cell lung cancer (NSCLC) who were treated with PD-1/PD-L1 immune checkpoint inhibitors were prospectively enrolled at ten tertiary medical centers between August 2020 and March 2021. Peripheral blood samples were obtained at baseline (before the start of therapy) and subsequently during treatment, according to predefined time points [30].

For each patient, whole blood was collected in EDTA tubes and processed within 24 h. Plasma was separated using a two-step centrifugation procedure and stored at −80 °C. Circulating cell-free DNA was isolated and quantified using the PicoGreen fluorescence assay (Thermo Fisher Scientific, Waltham, MA, USA).

For the normal control group, frozen plasma samples from ten healthy subjects were obtained from the Biobank of Ajou University Hospital.

Whole-exome libraries were constructed using the Agilent SureSelect Human All Exon V6 kit (Agilent Technologies, Santa Clara, CA, USA), and sequencing was performed on the Illumina NovaSeq platform (Illumina Inc., San Diego, CA, USA) with an average target coverage of 200×.

Sequencing reads were aligned to the hg19 reference genome using BWA-MEM, followed by standard post-processing steps such as duplicate marking and base quality recalibration. All procedures were consistent with those described in [4].

### 4.2. Raw Copy Number Ratio Quantification and Common Fluctuation Control

To measure the read count (RC) signal, the exonic regions were divided into variable-length bins, each consisting of 50 uniquely mappable bases, following the approach of Wabico and BIC-seq2 [31,32]. Because whole-exome sequencing (WES) targets only exonic regions, our analysis was restricted to uniquely mappable loci within these regions. For each bin, the total number of reads aligned to its positions was calculated as its RC value. The read count of bin *i* is denoted as RC(i).

Only autosomal chromosomes (1–22) were considered in the analysis, as sex chromosomes exhibit systematic differences in coverage between males and females. This binning process produced a total of 337,142 RC bins across the autosomes.

To estimate the relative copy number, where a diploid state is indicated by a value of 1 and values below or above 1 suggest deletion or amplification, respectively, RC(i) was transformed into normalized read counts (NRC) in several ways.

The simplest form, $NRC_{\mathrm{raw}}\left(i\right)$, was obtained by normalizing RC(i) by the sample-wide median:

$$NRC_{\mathrm{raw}}\left(i\right)=\frac{RC(i)}{\mathrm{Median}(RC)}.$$

It should be noted that $NRC_{\mathrm{raw}}\left(i\right)$ may still be influenced by technical artifacts such as sequencing bias or local coverage fluctuation.

When multiple control samples were available, the following equation was used to define the bias-corrected normalized read count, $NRC_{\mathrm{corrected}}\left(i\right)$, for the test sample:

$$NRC_{\mathrm{corrected}}\left(i\right)=\mathrm{Median}\;\left(\frac{NRC_{\mathrm{raw},\mathrm{test}}\left(i\right)}{NRC_{\mathrm{raw},{\mathrm{sample}}_{j}}\left(i\right)}\right).$$

Here, $NRC_{\mathrm{raw},\mathrm{test}}\left(i\right)$ represents the normalized read count at bin *i* for the test sample, while $NRC_{\mathrm{raw},{\mathrm{sample}}_{j}}\left(i\right)$ indicates the normalized read count at bin *i* for the *j*-th control sample. Systematic bias was reduced by taking the median of the normalized ratios across all control samples at each bin.

The steps for RC binning, normalization, and fluctuation correction used in this study were identical to those in our previous work [4].

### 4.3. GC Bias Correction

Although the previously described method effectively reduced inter-sample fluctuations, it did not fully eliminate spurious signals resembling CNAs. Residual false positives were further minimized using a LOESS regression model, as defined by the following equation:

$$NRC_{\mathrm{GC},\mathrm{corrected}}\left(i\right)=\mathrm{Median}\;\left(\frac{NRC_{\mathrm{corrected},{\mathrm{sample}}_{j}}\left(i\right)}{NRC_{\mathrm{fitted},{\mathrm{sample}}_{j}}\left(i\right)}\right),$$

where

$$NRC_{\mathrm{corrected},{\mathrm{sample}}_{j}}\left(i\right)=\frac{NRC_{\mathrm{raw},\mathrm{test}}\left(i\right)}{NRC_{\mathrm{raw},{\mathrm{sample}}_{j}}\left(i\right)},$$

and $NRC_{\mathrm{fitted},{\mathrm{sample}}_{j}}\left(i\right)$ is a LOESS regression model fitted with the GC ratio of bin *i* as the predictor and $NRC_{\mathrm{corrected},{\mathrm{sample}}_{j}}\left(i\right)$ as the response. The GC bias-corrected signal, $NRC_{\mathrm{GC},\mathrm{corrected}}\left(i\right)$, was defined as the median of the ratio $\frac{NRC_{\mathrm{corrected},{\mathrm{sample}}_{j}}\left(i\right)}{NRC_{\mathrm{fitted},{\mathrm{sample}}_{j}}\left(i\right)}$ across all control samples *j*, with a neutral copy number state of 1.

Copy number alterations in NRC profiles were segmented using the CBS algorithm [33] implemented in the DNAcopy R package 1.82.0 [34], with default parameters.

## 5. Conclusions

We established a GC bias-corrected cfDNA WES pipeline capable of reliably detecting CNAs and reproducing tissue-level genomic landscapes of NSCLC. The approach improved detection accuracy and consistency while reducing false positives. Although technical limitations such as low ctDNA fractions remain, the results highlight cfDNA WES as a feasible and complementary method for obtaining clinically relevant genomic information when tissue biopsy is limited or unavailable.

## Data Availability

The datasets presented in this article are not readily available because they contain sensitive clinical information and are restricted by patient privacy regulations. Requests to access the datasets should be directed to the corresponding author (oiny2000@kiom.re.kr) and may require approval from the Institutional Review Board.

## Abbreviations

The following abbreviations are used in this manuscript:

CBS	Circular binary segmentation
cfDNA	Cell-free DNA
CNA	Copy number alteration
CNV	Copy number variation
ctDNA	Circulating tumor DNA
ddPCR	Droplet digital polymerase chain reaction

LUAD	Lung adenocarcinoma
LUSC	Lung squamous cell carcinoma
NRC	Normalized read count
NSCLC	Non-small cell lung cancer
PD	Progressive disease
PFS	Progression-free survival
QC	Quality control
RC	Read count
SNV	Single-nucleotide variant
TCGA	The Cancer Genome Atlas
TF	Tumor fraction
TI wavelet transform	Translation-invariant wavelet transform
WES	Whole-exome sequencing
WGS	Whole-genome sequencing
